# Prioritising patients for publicly funded bariatric surgery in Queensland, Australia

**DOI:** 10.1038/s41366-024-01615-2

**Published:** 2024-08-22

**Authors:** Paul Scuffham, Megan Cross, Srinivas Teppala, George Hopkins, Viral Chikani, Katie Wykes, Jody Paxton

**Affiliations:** 1 Menzies Health Institute Queensland, Griffith University, Gold Coast, QLD Australia; 2 Royal Brisbane & Women’s Hospital, Queensland Health, Brisbane, QLD Australia; 3 Endocrinology, Princess Alexandra Hospital, Queensland Health, Brisbane, QLD Australia; 4 Healthcare Improvement Unit, Clinical Excellence Queensland, Queensland Health, Brisbane, QLD Australia

**Keywords:** Bariatric surgery, Health policy

## Abstract

**Objectives:**

This study reports the development and pilot application of the Bariatric Surgery Assessment and Prioritisation Tool (BAPT) for use in a public health system. The BAPT was designed as a patient prioritisation instrument to assess patients with excessive weight and type 2 diabetes suitable for bariatric surgery. We assessed whether the instrument successfully identified those who gained the greatest benefits including weight loss, diabetes remission, reduction in comorbidities, and health-related quality of life (HR-QoL).

**Methods:**

The BAPT instrument was applied to score 292 patients referred for bariatric surgery in Queensland between 2017 and 2020 based on their, body mass index, diabetes status, surgical risk (e.g. pulmonary embolism) and comorbidities (e.g. non-alcoholic steatohepatitis). These data were collected at referral and at 12-months post-surgery for 130 patients and stratified by BAPT scores. Outcomes included clinical and HR-QoL.

**Results:**

Patients’ BAPT scores ranged from 12 to 78 (possible range 2–98). Those with higher scores tended to be younger (*p* < 0.001), have higher BMI (*p* < 0.001) or require insulin to manage diabetes (*p* < 0.01). All patients lost similar percentages of body weight (20–25%, *p* = 0.73) but higher-scoring patients were more likely to discontinue oral diabetes medications (*p* < 0.001) and the improvement in glycated haemoglobin was four times greater in patients scoring 70–79 points compared to those scoring 20–29 (*p* < 0.05). Those who scored ≥ 50 on the BAPT were substantially more likely to obtain diabetes remission (57% vs 31%). BAPT scores of 40 and above tended to have greater improvement in HR-QoL.

**Conclusions:**

The BAPT prioritised younger patients with higher BMIs who realised greater improvements in their diabetes after bariatric surgery. Higher-scoring BAPT patients should be prioritised for bariatric surgery as they have a greater likelihood of attaining diabetes remission.

Excess weight is a significant risk factor in both chronic and acute disease. It contributes to the morbidity and mortality of multiple conditions, including cardiovascular disease [1], type 2 diabetes [2], cancer [3], liver and kidney disease [4], sleep apnoea [5] and depression [6]. The subsequent consequences for patient health and quality of life are significant. Patients with excess weight (body mass index (BMI) above 30 kg/m^2^) face up to 20% lower life expectancy [7] and 50% higher medical costs [8] than those in a normal weight range.

The severe obesity (BMI ≥ 35) rate in Australia has increased steadily and 31% of the population is now considered to have excess weight [9]. As excess weight contributes 8.4% of the country’s total burden of disease [9] and double health service utilisation rates, there is a strong incentive to improve accessibility to excess weight-related care in the public health system.

Bariatric surgery is the most effective treatment for severe obesity [10] and long-term studies confirmed its effectiveness in weight loss, reduction of obesity-related complications and improved quality of life and mortality [11–13]. However, bariatric surgery in Australia has been mostly performed in the private healthcare sector and remained largely inaccessible to many eligible patients due to socioeconomic and geographic inequality [14, 15].

Before 2020 [16], Australia had no central framework to guide provision of the surgery in the public sector, despite growing demand [17]. However, there were already publicly funded services for bariatric surgery available in Queensland in some areas under very limited conditions; access was not equitable and wait times were very long. Given the potential for demand to outstrip capacity and in response to a pressing need to establish a publicly funded bariatric surgery service with a statewide catchment, the Bariatric Surgery Initiative (BSI) commenced in Queensland in 2017. It aimed to develop an equitable assessment and prioritisation process for the state’s health department to provide bariatric surgery to eligible patients, regardless of their socioeconomic status, ethnicity or location. For this, the BSI implemented a state-wide central referral hub and developed the Bariatric Surgery Assessment and Prioritisation Tool (BAPT) to assess patients referred to the BSI and prioritise those likely to derive the greatest health benefit from the public resources invested. Referring clinicians provided data on patients’ clinical characteristics, pathology results, and other factors to the central hub to assess patient eligibility (Fig. 1) and priority for bariatric surgery (Table 1).

**Fig. 1:**
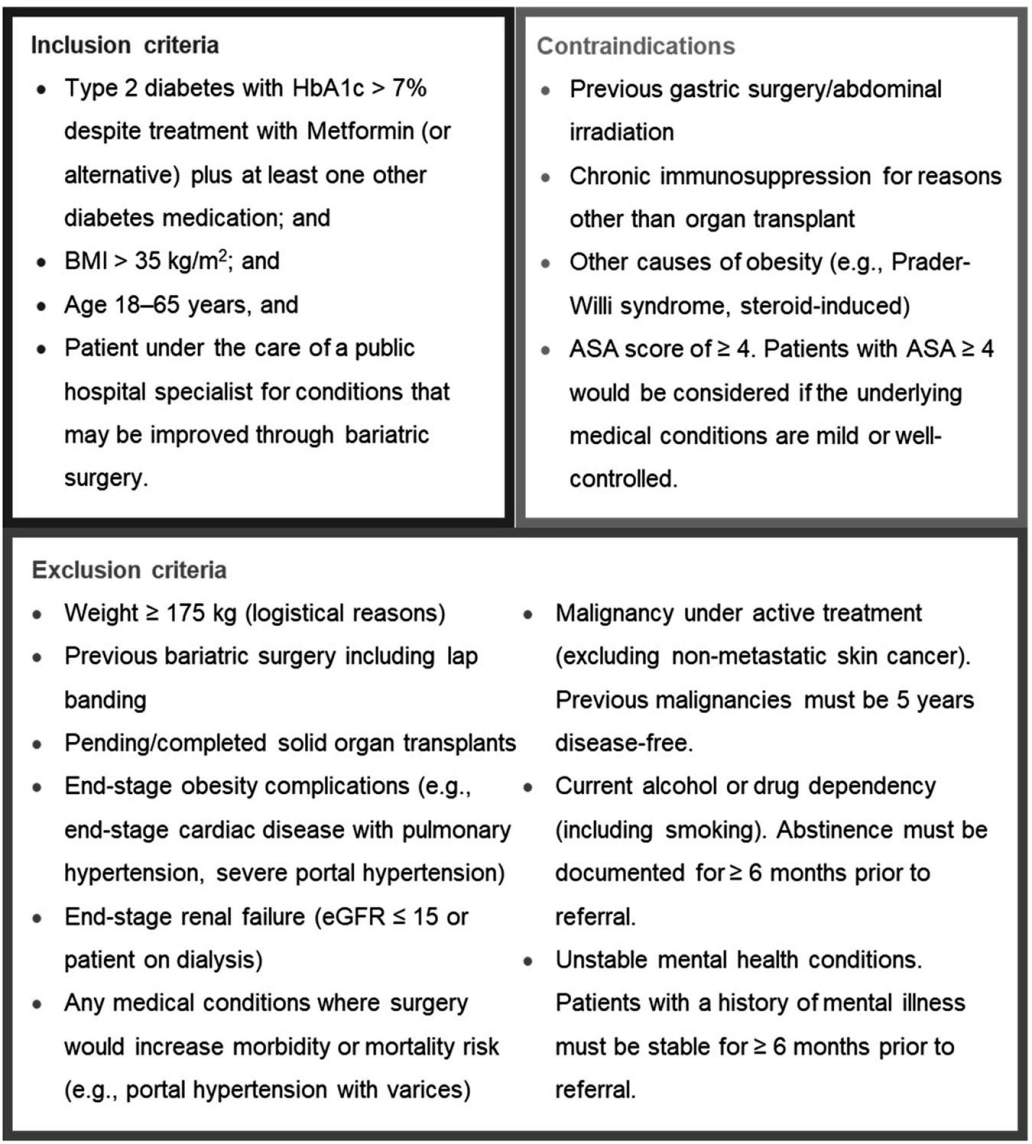
Inclusion and exclusion criteria of the bariatric surgery prioritisation tool. Note: “ASA, Australian Society of Anaesthetists. An ASA Class IV score is “A patient with incapacitating systemic disease that is a constant threat to life”. See: https://asa.org.au/preparing-for-your-anaesthetic/.

**Table 1:** Weighting criteria for scoring patients with the BAPT.

Criterion	Mean	SD	Normalised score	Rounded score	Maximum criterion score
Impact on life (comorbidities)					
Minimal impact	0	0	0		
Hypertension	8.72	4.88	1.96	2	
Dyslipidaemia	9.93	4.9	2.23	2	
Non-alcoholic steatohepatitis	13.36	4.54	3.01	3	
Albuminuria	15.43	5.52	3.47	3	
Joint pain	16.06	5.19	3.61	4	
Reproductive issues^a^	17.41	4.49	3.92	4	
Renal impairment (incl hyperfiltration)	23.43	5.11	5.27	5	
Obstructive sleep apnoea	30.28	7.46	6.81	7	30
Duration of diabetes					
Diabetes > 8 years	0	0	0	0	
Diabetes 4–8 years	11.92	8.1	11.92	12	
Diabetes < 4 years	19.92	14.42	19.92	20	20
BMI					
35 to < 40	0	0	0	0	
40 to < 45	8.63	4.9	8.63	10	
45 to < 50	11.58	4.77	11.58	13	
> 50	14.92	4.54	14.92	15	15
Age					
50 years and over	0	0	0	0	
30–49 years	8.76	3.66	8.76	10	
18–29 years	15.17	7.84	15.17	15	15
Surgical risk (risk factors: male, age 45 + , BMI ≥ 50 kg/m^**2**^, hypertension, risk of pulmonary embolism)					
12× risk (4 to 5 factors)	0	0	0	0	
5× risk (2 to 3 factors)	11.34	9.04	11.34	10	
Normal or slightly increased risk (0 or 1 factor)	19.7	11.62	19.7	20	20

^a^Reproductive issues include obstetric/gynaecological issues, infertility, male hypogonadism and erectile dysfunction.

## The bariatric surgery assessment and prioritisation tool

Accessing the BSI is a two-stage process. Patients must first meet a series of eligibility criteria for referral and they are then assessed and prioritised based on their likelihood of clinical benefit (i.e. predict better health outcomes) [18]. As the BSI is publicly funded, it used an approach that considered the views of the Australian general population to guide eligibility and assessment criteria. At the time of its inception, the debate regarding patient prioritisation focused on patient age and BMI. Participants in Australian citizens’ juries [19] considered bariatric surgery best provided to those aged 30–50 years with a BMI above 35 kg/m^2^. However, a large population discrete choice experiment [20] indicated that age was unimportant and that patients with higher BMI should be prioritised. In both studies, the presence of comorbidities was considered important. The priority of patients with higher BMI is further supported by cost-utility analyses demonstrating that cost savings are greatest in those with the highest pre-surgery BMI [21]. However, these background studies were insufficient for prioritising patients and subsequent clinical decisions. A “clinical and operational reference group” (CORG) of bariatric surgeons, primary care physicians, dietitians, endocrinologists, a health economist and policymakers from Queensland Health was established. The CORG reviewed existing evidence and adapted an earlier instrument from New Zealand [22] to form inclusion and exclusion criteria for bariatric surgery (Fig. 1) and create the BAPT to identify those who will benefit the most and should therefore be prioritised for surgery. We take a multifactorial approach to determining health benefits and who will “benefit the most” as there is no single composite measure for health benefits. We include clinical outcomes of weight loss, glycaemic control, diabetes medications, diabetes remission, obesity-related comorbidities and include a generic health-related quality of life (HR-QoL) measure.

This article reports on the development and pilot application of the BAPT to prioritise 292 patients referred to the BSI from September 2017 to August 2019, 212 of whom underwent surgery between December 2017 and August 2020. We consider the clinical characteristics of patients across the range of BAPT scores to determine whether the BAPT was able to prioritise those who should theoretically benefit the most. We then examine the patients’ clinical outcomes, stratified by their BAPT scores, and HR-QoL, to consider whether high-scoring patients achieved better outcomes.

## Methods

This study comprised 292 patients who consented to the use of their data for this research. This research involving human subjects and human data is in accordance with the Declaration of Helsinki. Ethics approvals for the evaluation were received from the Metro South Hospital and Health Service and Griffith University Human Research Ethics Committees on 20 September 2018 and 2 August 2019, respectively, with additional amendments approved on 18 January 2021 (Project HREC/18/QPAH/427). Written informed consent was obtained from all patients for their participation in the study, for release of their de-identified clinical data to Griffith University for the study and for them to undergo bariatric surgery.

### BAPT score development

Figure 1 presents the BSI’s inclusion and exclusion criteria and contraindications. Eligible patients are those aged 18–65 years with BMI > 35 kg/m^2^, poorly managed type 2 diabetes (i.e., glycated haemoglobin, HbA1c, >7% despite treatment) and potentially reversible comorbid conditions (e.g., sleep apnoea) [23].

The BAPT prioritisation system was developed using an adaptive Discrete Choice Experiment (DCE) using 1000minds^©^ software (www.1000minds.com) [24]. DCEs are a popular approach to identifying the relative importance of the criteria of interest based on stated preferences [25]. In this DCE, clinical experts in endocrinology [3], bariatric surgery [2], a General Practitioner, a dietitian, an anaesthetist and a nurse specialist participated. The task was to develop priority scores for the BAPT. Participants were given a choice task with two descriptions of health states and were forced to choose which of the two (hypothetical) patients they would prioritise for bariatric surgery. The characteristics of the health states were then varied and the respondent was presented with a choice of two new health state descriptions. This process was repeated until all possible permutations had been ranked. 1000minds^©^ uses the Potentially All Pairwise RanKings of all possible Alternatives (PAPRIKA) method [26], which capitalises on the “transitivity” theorem, minimising the choice tasks presented to each participant. That is, if a participant prefers choice A to B, and B to C, then by transitivity A is preferred to C, and the participant is not presented with this choice.

Thus, the DCE is adaptive in the sense that the sequence and set of choice tasks presented to each participant depend on their earlier answers. Then, from each participant’s pairwise rankings of the choice tasks, linear programming embedded in 1000minds^©^ is used to derive weights of the relative importance for the levels on each criteria. For technical details see Hansen and Ombler [26]; for a recent review see Sullivan et al [27].

We specified five attributes, each of which contained various “levels”, as shown in Table 1. The attributes were: impact on potentially reversible conditions (9 levels), duration of diabetes (3 levels), age group (4 levels), BMI category (3 levels) and surgical risk (3 levels). Overall, there was a maximum of 9 × 3 × 4 × 3 × 3 = 972 possible health states for the five BAPT attributes. The 1000Minds^©^ software reduced the mean number of choice tasks to a more-manageable 24 choice decisions (e.g., Supplementary Fig. S1). From the respondents’ decisions, the software calculated weights for each level within a criterion as well as the overall criterion weight. Scores were normalised across criteria to provide relative preference weights, which are then scaled such that the sum of all criteria ranges from 0 (least preferred) to 100 (most preferred); the possible range for the BAPT was 2–98 with higher scores indicating those who are predicted to benefit the most.

Eighteen clinical experts in excess weight management across Queensland were invited to participate; nine completed the experiment. The BSI CORG reviewed the results, deliberated, and rounded the results due to the small sample size and for ease of scoring. This produced the final BAPT scoring system (Table 1): impact on potentially reversible conditions (30 points), expected benefit from controlling diabetes (20 points), age (15 Points), BMI (15 points) and surgical risk (20 points).

### Patient referral and surgical pathway

The full BSI referral and surgical pathway were reported earlier [28]. Briefly, patients were referred to the BSI by specialist outpatient clinics and BAPT scores were calculated for each referral and intended to be used to prioritise patients, especially when demand exceeded surgical capacity. It is important to note that all patients were assessed by an expert multidisciplinary medical team prior to surgery. Thus, while the BAPT aimed to flag patients of high priority, progression to surgery depended on the assessment team’s decision regarding the best pathway for each patient: not all patients with high scores underwent bariatric surgery and some who did have surgery had comparatively lower scores. At the time of writing, 42 patients were excluded from the service by contraindications/exclusion criteria [28], while 43 others remained under review due to COVID-19-related interruptions.

Eligible patients underwent bariatric surgery at one of two hospitals in Brisbane. Follow-up data on patients’ weight, BMI, diabetes and comorbidities were collected at 12 months post-surgery and the full clinical outcomes were reported [28].

### Analysis of BAPT scores and patient outcomes

The outcomes of interest include body weight, BMI, HbA1c, diabetes medications, diabetes remission defined as HbA1c < 6.5% and no diabetes medications [29], comorbidities and HR-QoL measured using the AQoL-4D scored using the Australian value set [30] at 12 months post-surgery. The AQoL-4D has four domains pertaining to independent living, relationships, senses, and mental health and a summary score on a scale of 0.0 = dead and 1.0 = best possible health. These data were stratified by patients’ BAPT scores to consider whether higher-scoring patients had better outcomes. A minimum important difference in AQoL-4D score is 0.06 [31]. Patients’ duration of diabetes before referral was also considered. To validate the prioritisation of younger patients and those with higher BMI, the outcome data were also stratified using three cut-points for age (50, 55, and 60 years) and BMI (40, 45, and 50 kg/m^2^) – for example, 18–49 vs 50 and over, 18–54 vs 55 and over, and 18–59 vs 60 and over.

### Statistical analysis

Descriptive statistics (frequencies, mean ± SD) were calculated for clinical measures at referral (pre-surgery) and 12 months post-surgery and were evaluated for significant differences over that period. We used chi-square tests to access relationships between nominal or ordinal dependent variables across categories, and one-way ANOVA to examine differences over the means of interval dependant variables. All analyses were performed using SAS (v9.4; SAS Institute, NC, USA) or IBM SPSS (v27.0, IBM Corp., NY, USA).

## Results

### Patient cohort

The BSI calculated BAPT scores for 292 referred patients. At referral, the cohort was on average (mean ± SD) 52 ± 8.7 years old and 57.1% female, with a mean BMI of 46.1 ± 7.0 kg/m^2^; 21.9% were Aboriginal or Torres Strait Islanders. The average HbA1c was 8.77 ± 1.5%; 99.7% of patients required oral medications for diabetes. All patients reported comorbidities, the most common of which were hypertension (86.3%), dyslipidaemia (85.2%) and sleep apnoea (66.0%). These and further patient characteristics at referral are stratified by BAPT score in Table 2 and detailed in the supplementary material.

**Table 2:** Characteristics of patients referred to the BSI, stratified by BAPT score.

BAPT score	10–19	20–29	30–39	40–49	50–59	60–69	70–79	*p*-value
*N*	13	34	77	74	56	27	11	
Age, years	58.0 ± 4.0	55.4 ± 6.2	54.7 ± 6.7	53.2 ± 9.0	49.2 ± 8.1	45.2 ± 10.7	40.3 ± 6.8	< 0.001*
Sex								0.13
Males	8 (61.5%)	16 (47.1%)	35 (45.5%)	34 (45.9%)	23 (41.1%)	8 (29.6%)	1 (9.1%)	
Females	5 (38.5%)	18 (52.9%)	42 (54.5%)	40 (54.1%)	33 (58.9%)	19 (70.4%)	10 (90.9%)	
Weight, kg	115 ± 22.2	119.0 ± 21.3	125.6 ± 19.9	133.3 ± 20.0	137.5 ± 19.1	142.9 ± 14.5	134.0 ± 17.4	< 0.001*
BMI, kg/m^2^	38.6 ± 5.6	41.3 ± 5.5	44.3 ± 5.6	46.7 ± 6.6	49.1 ± 6.7	50.7 ± 6.9	49.6 ± 7.7	< 0.001*
Diabetes								
HbA1c, %	8.2 ± 0.6	9.2 ± 1.7	8.7 ± 1.2	8.6 ± 1.3	8.5 ± 1.4	9.4 ± 2.0	9.5 ± 1.7	0.1
Oral medications	13 (100%)	33 (100%)	75 (98.6)	72 (97.3%)	56 (100%)	26 (96.3%)	11 (100%)	0.7
Insulin	11 (84.6%)	25 (75.8%)	58 (76.2%)	47 (63.5%)	31 (55.3%)	13 (48.1%)	2 (18.2%)	< 0.001*
Duration of diabetes								
Less than 4 years	0 (0.0%)	0 (0.0%)	0 (0.0%)	4 (5.4%)	10 (17.9%)	11 (40.7%)	9 (81.8%)	< 0.001*
4–8 years	0 (0.0%)	0 (0.0%)	14 (18.2%)	18 (24.3%)	33 (58.9%)	15 (55.6%)	2 (18.2%)	< 0.001*
Longer than 8 years	13 (100%)	34 (100%)	63 (81.8%)	52 (70.3%)	13 (23.2%)	1 (3.7%)	0 (0.0%)	< 0.001*
Comorbidities								
Hypertension	13 (100%)	31 (96.9%)^a^	67 (89.3%)^b^	68 (93.2%)^c^	44 (81.5%)^d^	23 (95.8%)^e^	6 (54.5%)^f^	
Dyslipidaemia	13 (100%)	28 (82.4%)	71 (92.2%)	62 (83.8%)	48 (85.7%)	19 (70.4%)	8 (72.7%)	
Sleep apnoea	3 (30%)	8 (47.1%)^g^	34 (65.4%)^h^	43 (74.1%)^i^	37 (82.2%)^j^	16 (80.0%)^k^	7 (100%)^l^	
Joint pain	5 (38.5%)	14 (41.2%)	36 (46.8%)	53 (72.6%)^c^	30 (54.5%)	19 (70.4%)	5 (45.5%)	
NASH	1 (7.7%)	4 (11.8%)	9 (11.8%)	9 (12.2%)	11 (19.6%)	1 (3.7%)	1 (9.1%)	
Renal impairment	0 (0.0%)	3 (8.8%)	7 (9.1%)	8 (11.0%)	3 (5.4%)	1 (3.7%)	0 (0.0%)	
Reproductive issues	0 (0.0%)	6 (18.2%)^m^	9 (11.7%)	25 (33.8%)	9 (16.1%)	8 (29.6%)	4 (36.4%)	

Data are presented as mean ± standard deviation or *N* (%). *N* = 292 patients.

*Statistically significant, *p* < 0.05.

^a^*N* = 32, ^b^*N* = 75, ^c^*N* = 73, ^d^*N* = 54, ^e^*N* = 24, ^f^*N* = 11, ^g^*N* = 17, ^h^*N* = 52, ^i^*N* = 58, ^j^*N* = 45,

^k^*N* = 20, ^l^*N* = 7, ^m^*N* = 33.

### BAPT scores and prioritised patients

**Fig. 2:**
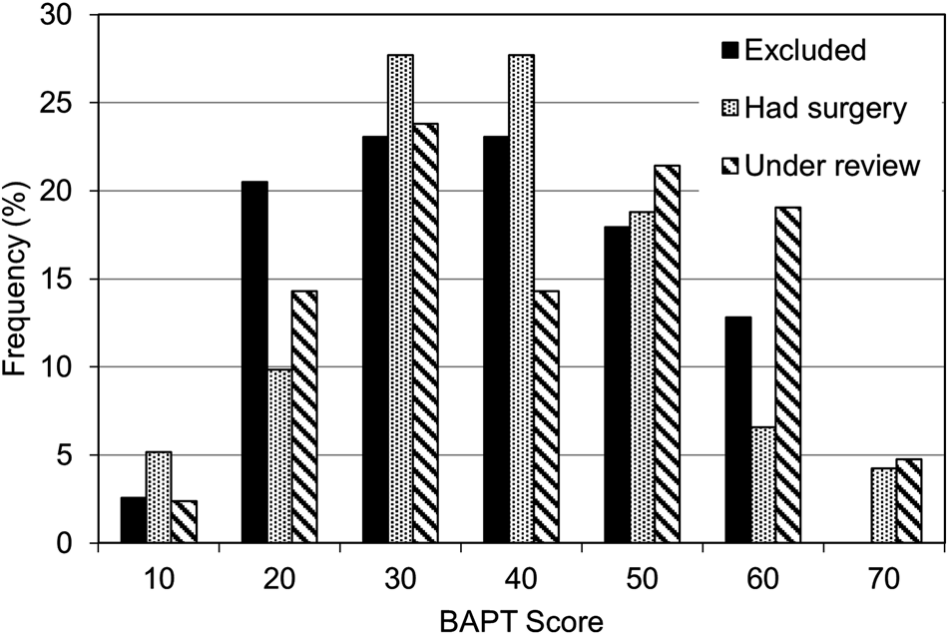
Distribution of BAPT scores. Percentage of BAPT scores for patients who were excluded from surgery (solid columns), those who underwent surgery (column with dots) and those waiting for surgery (hatched column).

The BAPT scores of 292 patients referred to the BSI were normally distributed between 12 and 78 (mean ± SD: 43 ± 14). The distribution of BAPT scores was similar across patients who had surgery (42.5 ± 13.2), those who remained under review (47.1 ± 13.5) and those excluded from the service (42.0 ± 14.2) (Fig. 2).

Referred patients were 22–66 years old and younger patients had higher BAPT scores overall (Supplementary Fig. S2A). For example, the average age of patients scoring 20–29 was 55.4 ± 6.2 years; that of patients scoring 60–69 was 45.2 ± 10.7 years (*p* < 0.001; Table 2). Nevertheless, 91% of the cohort that progressed from referral to surgery was older than 40 years and the average age of patients having surgery was 52.2 ± 8.4 years [28].

Patients with higher BMI had higher BAPT scores (*p* < 0.001). For example, patients scoring 20–29 had an average BMI of 41.3 ± 5.5 kg/m^2^, while those scoring 60–69 had an average BMI of 50.7 ± 6.9 kg/m^2^ (Table 2). Further, a greater proportion of those with higher scores had a BMI ≥ 40 kg/m^2^; (category III obesity, Supplementary Fig. S2C).

The average HbA1c across all BAPT scores was similar at referral. Although the lowest average HbA1c was seen in low-scoring patients (BAPT 10–19; 8.2 ± 0.6%) and the highest occurred in high-scoring patients (BAPT 70–79; 9.5 ± 1.7%), there was no correlation between HbA1c and BAPT score (Spearman’s ρ = –0.03; *p* = 0.73). However, there was a significant association between BAPT score and diabetes medication use pre-surgery. The proportion of patients in each BAPT group who required insulin decreased with increasing BAPT score (*p* < 0.001). For example, 75.8% of patients scoring 20–29 required insulin, compared to 48.1% of those scoring 60–69 (Table 2). Additionally, BAPT scores were higher for those with a shorter duration of diabetes before referral. For example, 82% of those scoring above 70 had diabetes for less than four years, while those with diabetes for longer than eight years populated the lower-scoring groups (Table 2).

Patients are also scored on the presence of selected comorbidities (Table 2). These varied widely between BAPT score ranges and while the proportion of patients reporting weight-related joint pain and sleep apnoea increased at higher BAPT scores (joint pain: 38.5% at BAPT 20–29 v. 70.4% at BAPT 60–69; sleep apnoea: 47.1% at BAPT 20–29 v. 80% at BAPT 60–69), there were no trends regarding other comorbidities.

### Clinical outcomes post-surgery

**Fig. 3:**
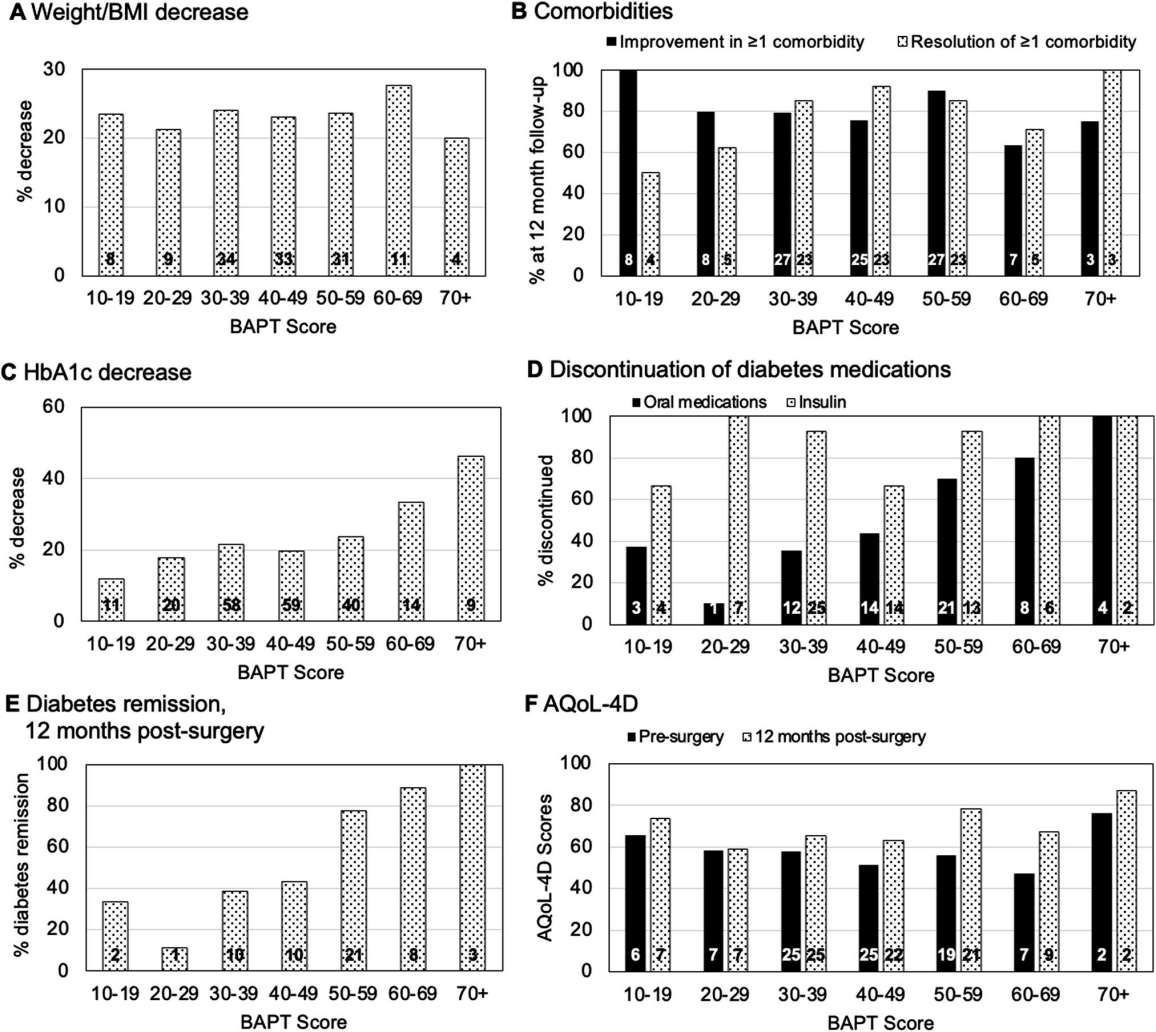
Changes in patients’ weight, diabetes and comorbidities from referral to 12 months post-bariatric surgery, stratified by BAPT score. Each panel displays the clinical and health outcomes at 12 months post-surgery compared with pre-surgery measures stratified by BAPT score. **A** Percentage decrease in weight (same as percentage decrease in BMI). **B** Improvement in at least 1 comorbidity (solid column) and resolution of at least 1 comorbidity (hatched column). **C** Percentage decrease in HbA1c. **D** Percentage discontinuing oral diabetes medications (solid column) and insulin (hatched column). **E** Percentage achieving remission of diabetes. **F** AQoL-4D score at pre-surgery and post-surgery (higher values indicate better quality of life). The numbers within each column are the sample with data reported in each category.

Of the 212 patients that had surgery, 130 patients had reached a minimum of 12 months post-surgery before data collection for the study stopped; this was partly due to the COVID-19 pandemic interruption, however, the BSI continued. There were no losses to follow up. The data for the 130 patients at 12 months post-surgery were compared to their pre-surgery values and then stratified by their BAPT scores to consider whether those with higher scores had better outcomes (Fig. 3).

#### Weight loss

Patients with higher pre-surgery weight and BMI had higher BAPT scores. However, there was no significant difference in the percentage of weight lost (i.e. % BMI decrease) by patients with different BAPT scores (*p* = 0.73; Fig. 3A).

#### Diabetes

Differences were noted between BAPT scores regarding diabetes-related outcomes. While there was no significant difference in HbA1c values pre-surgery (*p* = 0.36), higher-scoring patients had lower HbA1c at 12 months (*p* < 0.01) and had a significantly greater decrease in HbA1c (*p* < 0.01; Table 2). For example, those with BAPT scores of 20–29 had an average pre-surgery HbA1c of 8.2 ± 0.7%, which decreased to 7.3 ± 1.8% post-surgery — an 11.8% improvement (Table 2, Fig. 3C). In contrast, the pre-surgery average HbA1c of patients with BAPT scores of 70–79 was 9.5 ± 1.8%, decreasing to 5.3 ± 0.4% — a 46.4% improvement (Table 2, Fig. 3C), approximately four times greater than the BAPT 20–29 group.

Differences were also noted in patients’ diabetes medications after surgery. All patients required oral medications, insulin, or both, at referral. Post-surgery, 60–80% of patients across all BAPT scores discontinued insulin treatment (Fig. 3D), with no trend regarding BAPT score. However, a greater proportion of higher-scoring patients discontinued oral medications after 12 months (*p* < 0.001; Fig. 3D). Overall, using the definition of remission of diabetes of HbA1c below 6.5% (48 mmol/mol) in the absence of glucose lowering medications [32], 39.3% of the sample obtained diabetes remission; this increased with BAPT score (*p* = 0.327; Fig. 3E) with those who scored 50 or more were substantially more likely to obtain remission (57% vs 31%).

#### Comorbidities

The degree of heterogeneity in patients’ comorbidities complicated the comparison of their post-surgical outcomes. This was further challenged by delays due to the COVID-19 pandemic, which meant that fewer patients had 12-month follow-up data. For simplicity, we considered the proportions of patients within each BAPT score range who completed 12-month follow-up and who reported improvement in at least one condition reported at referral (Fig. 3B). Follow-up completion ranged from 29.4% to 61.5% and at least 80% of patients reported improvement in at least one comorbidity and 66% reported resolution of at least one comorbidity. However, there was no trend in the percentage of patients with improvement based on the BAPT score at recruitment.

#### Surgical risk

There were 32 adverse events (AEs) reported in 28 patients across the BAPT categories (Supplementary Table S1). The most common were being readmitted within 28 days (*n* = 15), then nausea/vomiting (*n* = 7). The number of patients with an AE, and the number of AEs were independent of the total BAPT score (*p* = 0.962 and *p* = 0.894 respectively). Examining the BAPT domain score for surgical risk, there was no significant pattern to either the number of people with an AE or the number of AEs (*p* = 0.584 and *p* = 0.559).

#### Age and BMI cut-points

To consider whether younger patients benefitted more than their older peers, we examined health outcomes after surgery using cut-points at 50, 55 and 60 years old (Table 3). A significant difference was noted in the 18–49-year-olds compared to those older than 50 years, where the younger group achieved a greater decrease in average HbA1c (27.3% v. 20.1%, *p* < 0.01; Table 3).

**Table 3:** Comparison of patient outcomes with age and BMI cut-offs.

Age/BMI	*N*	Decrease in BMI, %	Improvement in HbA1c, %	No diabetes meds post-surgery, *n* (%)
Age 18–49	45	24.6 ± 9.9	27.3 ± 18.4	26 (59.1%)
Age ≥ 50	85	22.9 ± 8.4	20.1 ± 13.6	37 (43.5%)
*p*-value		0.3	0.01*	0.07
Age 18–54	73	24.9 ± 9.7	24.7 ± 16.3	38 (52.8%)
Age ≥ 55	57	21.8 ± 7.7	19.7 ± 14.6	25 (43.9%)
*p*-value		0.05	0.08	0.3
Age 18–59	100	23.7 ± 9.3	23.9 ± 15.8	48 (48.5%)
Age ≥ 60	30	22.9 ± 7.6	17.9 ± 14.6	15 (50.0%)
*p*-value		0.7	0.07	0.9
BMI < 40	50	22.3 ± 7.1	21.8 ± 14.2	16 (32.0%)
BMI ≥ 40	80	24.3 ± 9.9	22.9 ± 16.6	47 (59.5%)
*p*-value		0.2	0.7	0.002*
BMI < 45	83	22.3 ± 7.9	22.3 ± 15.2	36 (43.4%)
BMI ≥ 45	47	25.6 ± 10.3	22.7 ± 16.8	27 (58.7%)
*p*-value		0.04*	0.9	0.1
BMI < 50	111	23.4 ± 9.0	23.4 ± 15.3	52 (47.3%)
BMI ≥ 50	19	24.4 ± 8.8	17.1 ± 17.1	11 (57.9%)
*p*-value		0.6	0.1	0.4

*Statistically significant *p* < 0.05.

Percentage improvement/decrease was calculated at 12-month post-surgery follow-up relative to referral.

Similar analyses of the results with BMI cut-points of 40, 45 and 50 kg/m^2^ found a significant difference in BMI decrease at 12 months in patients with a starting value above 45 kg/m^2^. Their average BMI decreased by 25.6 ± 10.3%—a 3.3% greater decrease than the 22.3 ± 7.9% achieved by patients with BMI below 45 kg/m^2^ (Table 3). A lower BMI cut-point of 40 kg/m^2^ found differences in the proportion of patients able to discontinue medications for diabetes by 12 months post-surgery. Similarly, in all three analyses, a greater proportion of the higher-BMI group discontinued all diabetes-related medications, though the difference was only statistically significant with a cut-point of 40 kg/m^2^ (59.5% v. 32.0%, *p* < 0.01). No significant differences in BMI or diabetes care were observed with a cut-point of 50 kg/m^2^.

#### Health-related quality of life

Those with low BAPT scores tended to have better HR-QoL pre-surgery than those with high BAPT scores (Fig. 3F). For example, the AQoL-4D scores for those with a BAPT score below 40 vs 40 and above were 0.583 ± 0.242 vs 0.529 ± 0.217; *p* = 0.379. The change in AQoL-4D scores at 12 months post-surgery tended to be greater for those with higher BAPT scores (e.g. mean change in AQoL-4D: BAPT < 40 = 0.058 ± 0.277 vs BAPT 40 and above = 0.174 ± 0.235; *p* = 0.095); this is a 10% vs 33% improvement in HR-QoL. There was no statistically significant difference in AQoL-4D scores across BAPT categories (*p* = 0.184); the BAPT category 50–59 was the only category with a significant improvement in AQoL-4D score (0.200 ± 0.246; *p* = 0.032). The change in AQoL-4D scores for BAPT categories 40 and above, plus the BAPT 10–19, met the minimum important difference of 0.06 [31] (Supplementary Table S2).

## Discussion

Provision of bariatric surgery as a publicly funded health service requires processes that ensure equitable access alongside the strategic allocation of resources to maximise value for patients and manage the service’s long-term sustainability amid high demand. The BSI was open to referrals from specialists (and Aboriginal Community Controlled Health Organisations for Aboriginal patients) across Queensland. It implemented a central referral hub to standardise patient processing and maintain a robust, consistent referral pathway to ensure that surgery was offered to patients likely to receive the greatest clinical benefit while keeping patient assessment fair, consistent and maximising the value necessitated for the development of a comprehensive instrument tailored for the specific circumstances of patients with severe obesity. The BAPT was based on factors likely to influence the health outcomes of bariatric surgery (i.e., young age, high BMI, co-morbidities) [33] and, to the best of the authors’ knowledge, was the only such instrument in Australia before 2020. Bariatric surgery for patients with excess weight and diabetes was shown to be highly cost-effective [21] and the BAPT is one approach to prioritising patients for surgery. We previously assessed equity in patient access to the BSI program [34]. This paper builds on those efforts by examining the pilot application of the BAPT and whether the patients it prioritised realised greater health.

### Who scored high — and did high-scoring patients have better outcomes?

As designed, the BAPT prioritised those for surgery as younger, higher BMI and those who had diabetes for a shorter period. High-scoring patients were also less likely to rely on insulin to manage diabetes pre-surgery, which may reflect the duration or severity of their condition. The percentage of those who obtained diabetes remission and the improvements in quality of life were substantially greater for those who scored 50 or more on the BAPT compared with those who scored lower. However, low surgical-risk patients were assigned a high score in the surgical risk domain (20 points) and moderate-risk patients were assigned 10 additional points but AEs were distributed across all BAPT categories including within the BAPT risk-domain categories. Thus, the BAPT was poor at predicting AEs.

### Body mass

We note that patients with higher scores did have greater percentage decreases in weight compared with lower-scoring patients. Nevertheless, supplementary analyses found that patients with BMI above 40 kg/m^2^ had a greater percentage decrease in weight compared to those with a BMI below 40. Similarly, patients younger than 50 years benefitted most in terms of their diabetes. This suggests that the BAPT is indeed prioritising those age and BMI groups likely to benefit most. The fact that patients with higher BAPT scores did not lose more weight is likely a result of the BAPT instrument’s multifactorial composition, which allows patients who are older or leaner to still score high if they have severe comorbidities or diabetes that could benefit significantly from bariatric surgery.

### Diabetes

Higher-scoring patients derived greater benefits to their diabetes and were more likely to discontinue insulin or oral medications, especially those with a BAPT score of 50 or greater. The BAPT prioritises patients who have had type 2 diabetes for short periods and patients without insulin requirement because the short duration of diabetes and relatively preserved pancreatic function pre-surgery are the strongest predictors of diabetes remission post-surgery [35, 36]. This association was confirmed in the current sample and is the most likely explanation for the diabetes benefits derived from surgery in higher-scoring groups, particularly since current evidence suggests that patients’ starting BMI does not affect their HbA1c after bariatric surgery [37] and diabetes-related benefits are realised before significant weight loss occurs [38].

### Instruments prioritising bariatric surgery patients

At its inception in 2017, the BAPT was unique as the only instrument in Australia for prioritising bariatric surgery patients and there was no national framework for the public system. The Australian and New Zealand Metabolic and Obesity Surgery Society (ANZMOSS) and National Association of Clinical Obesity Services (NACOS) have since published a National Framework for Clinical Obesity Services [16], which includes patient prioritisation criteria; that framework has many similarities with the BAPT regarding inclusion/exclusion criteria, and comorbidities, and also recommends use of a “central referral hub” which increases access to regional patients and goes a long way in addressing inequities.

Several other instruments have been developed to predict the outcomes of BS. The study by Sesconetto (2023) [39], provides a meta-analysis and tests the performance (cut-points, sensitivity, specificity, positive and negative likelihood ratios and AUC) of five predictive instruments—the ABCD [40], DiaRem [41], Ad-DiaRem [42], IMS [43], and DiaBetter [44]. The author concludes that the IMS is most reliable when T2DM remission is the primary outcome. Compared with the BAPT, the BAPT was to prioritise patients for BS based on their ability to benefit the most, including T2DM remission. Moreover, the scales used by the other instruments differ considerably and a direct comparison with the BAPT developed was not possible in this study.

The present study sought to focus on prioritising those who would benefit the most from bariatric surgery. However, benefits from bariatric surgery are multi-dimensional and multifaceted. Unfortunately, to date, there is no single instrument that provides a composite measure of health benefits from bariatric surgery. Diabetes remission has been used as a proxy for clinical outcomes in several studies [40, 43, 45–74] and was included as one of several outcome measures in this study. The AQoL-8D is a generic health-related quality of life instrument but factors other than obesity, diabetes or bariatric surgery can affect overall HR-QoL. Hence, HR-QoL and any other single health or clinical outcome measure was not, and cannot be, used as the sole entity to measure “health benefits”.

The BAPT was developed with clinicians making forced trade-offs between patient characteristics to develop the weights for each category and feature. This is an important concept in economics, that is, there is always an opportunity cost and by forcing trade-offs, the marginal “value” of benefits from treating one patient’s characteristics over another can be determined. No other patient priority tool for bariatric surgery has taken this approach. Future studies, possibly with modifications to the BAPT, should consider having sufficient patient numbers with 12 or 24 months of follow-up data to undertake statistical comparisons of the BAPT with other instruments, especially instruments that use diabetes remission as the primary outcome, to determine the predictive value and external validity of the BAPT.

### Limitations

A small sample of clinical experts participated in the choice experiment to develop weights for the BAPT criteria; however, each clinician provided a wealth of data. The BAPT uses BMI as a broad indicator of severe obesity and acknowledges that it cannot capture the nuances of individual patients’ conditions (e.g., body composition). Further, the BMI that indicates greater health risk also varies between ethnic groups [75]; the BAPT does not consider ethnicity so patients’ scores may not reflect their clinical needs. However, BMI is one of several factors assessed by the BAPT, and the inclusion of obesity-related comorbidities is likely to capture the health consequences of excess weight where BMI is insufficient.

Fewer referrals were received during the first six months of the BSI program as clinician awareness of the Initiative grew. Patients with lower BAPT scores who may not have been prioritised under busier circumstances received surgery despite their low scores.

We note that comorbidity data, particularly the less common conditions (e.g., NASH), had relatively small samples at 12-month follow-up. Further, BSI patients underwent either sleeve gastrectomy or Roux-en-Y gastric bypass. Although the choice of procedure may influence outcomes in the long term [76], the potential effect of the different procedures on outcomes was not controlled for in this analysis, as procedures were selected on an individual basis following consultation with the multidisciplinary medical team.

Although there were trends towards patients with higher BAPT scores having greater improvements in HR-QoL, the small numbers of patients in each BAPT category and incomplete data limited the ability to be definitive about improvements in some measures including HR-QoL and the ability to differentiate between low, moderate and high surgical risk.

## Conclusion

Bariatric surgery is currently the most effective treatment for severe obesity and its provision as a publicly funded service has the potential to benefit both the health of patients and the sustainability of the health care system. Ensuring maximum benefit for both requires careful resource allocation and patient prioritisation. The BAPT was the first instrument in Australia to assess and score patients based on the likelihood of benefits from bariatric surgery. In this pilot study, the BAPT successfully prioritised those who had diabetes for a shorter period and younger patients, who realised greater improvements in their diabetes, and those with higher BMI. Most notably, patients’ diabetes improvement increased with their BAPT scores, as did their health-related quality of life 12 months post-surgery, indicating that the instrument prioritised those likely to gain greater benefits. Overall, these initial findings indicate that the BAPT is functioning as designed as a patient prioritisation instrument for bariatric surgery.

## Supplementary information

Prioritising Patients for Publicly Funded Bariatric Surgery in Queensland, Australia - Supplementary material

## Data Availability

The datasets generated for the BAPT are available from the corresponding author on reasonable request. The clinical data used is government-owned. An application for data access under the Public Health Act 2005 must be approved by the data custodian and the Director General of Queensland Health. To access the Statewide Bariatric Surgery Data Collection email the request to the Executive Director, Healthcare Improvement Unit at HAAT@health.qld.gov.au. For further information: https://www.health.qld.gov.au/hsu/pha.
