# Supplementary material for: Prioritising patients for publicly funded bariatric surgery in Queensland, Australia
Source: Int J Obes (Lond). 2024 Aug 22;48(12):1748–57. doi: 10.1038/s41366-024-01615-2 (PMC11584382; doi:10.1038/s41366-024-01615-2)
Supplement: Supplementary file 1 — Prioritising Patients for Publicly Funded Bariatric Surgery in Queensland, Australia - Supplementary material [file 41366_2024_1615_MOESM1_ESM.docx]

# Prioritising Patients for Publicly Funded Bariatric Surgery in Queensland, Australia

# Supplementary material


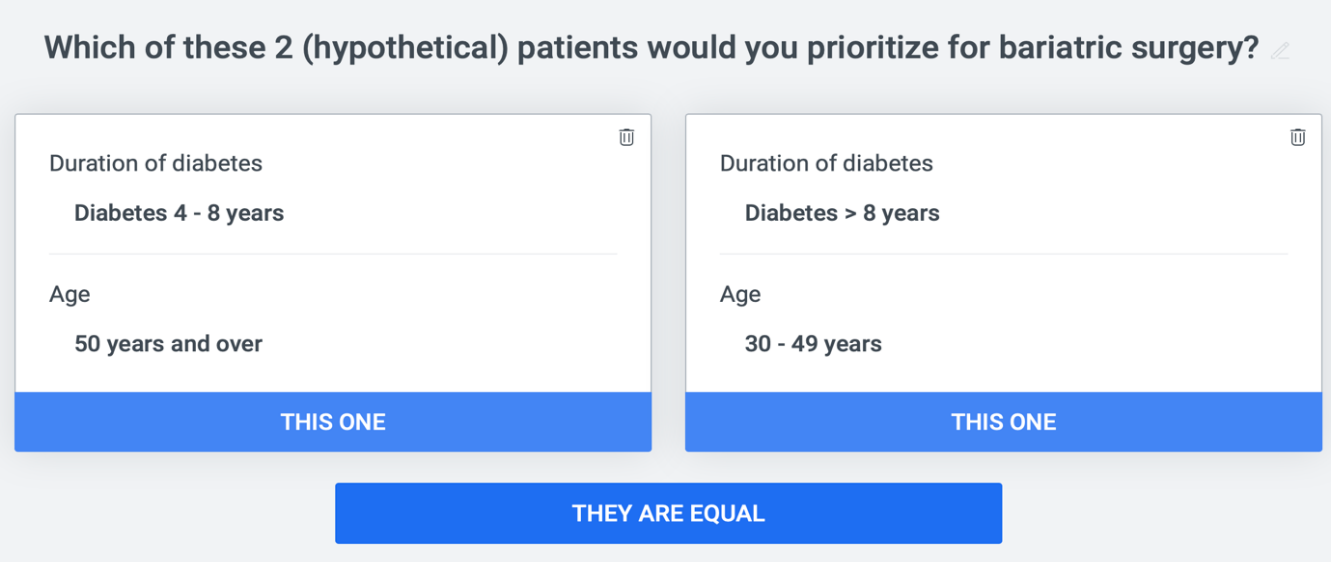

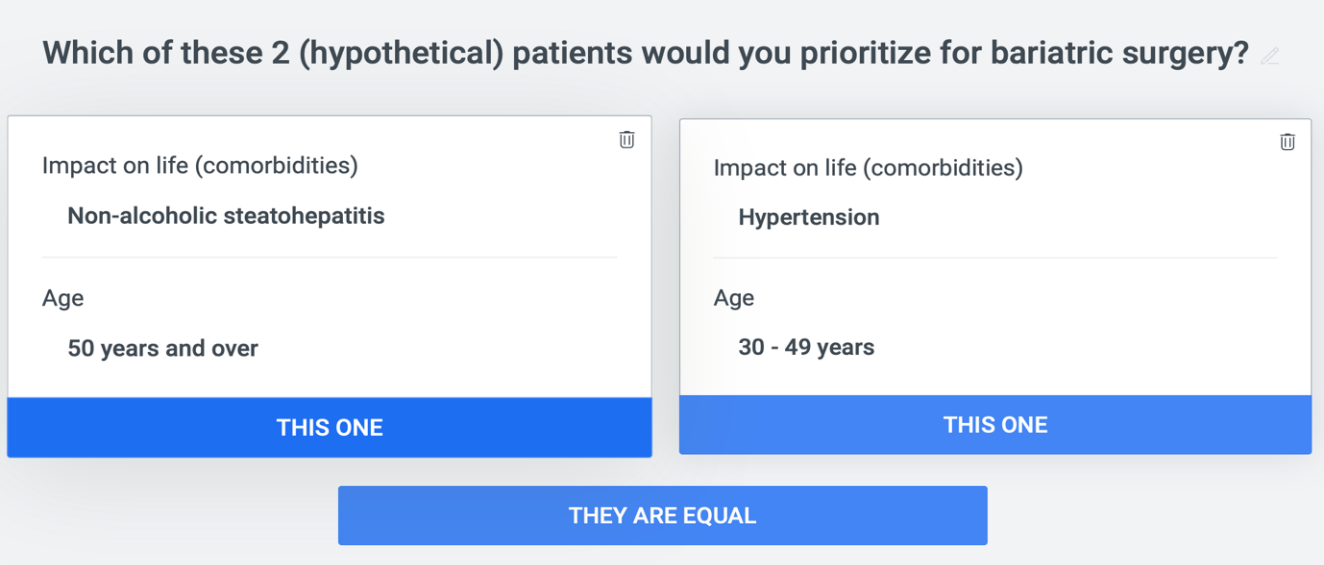

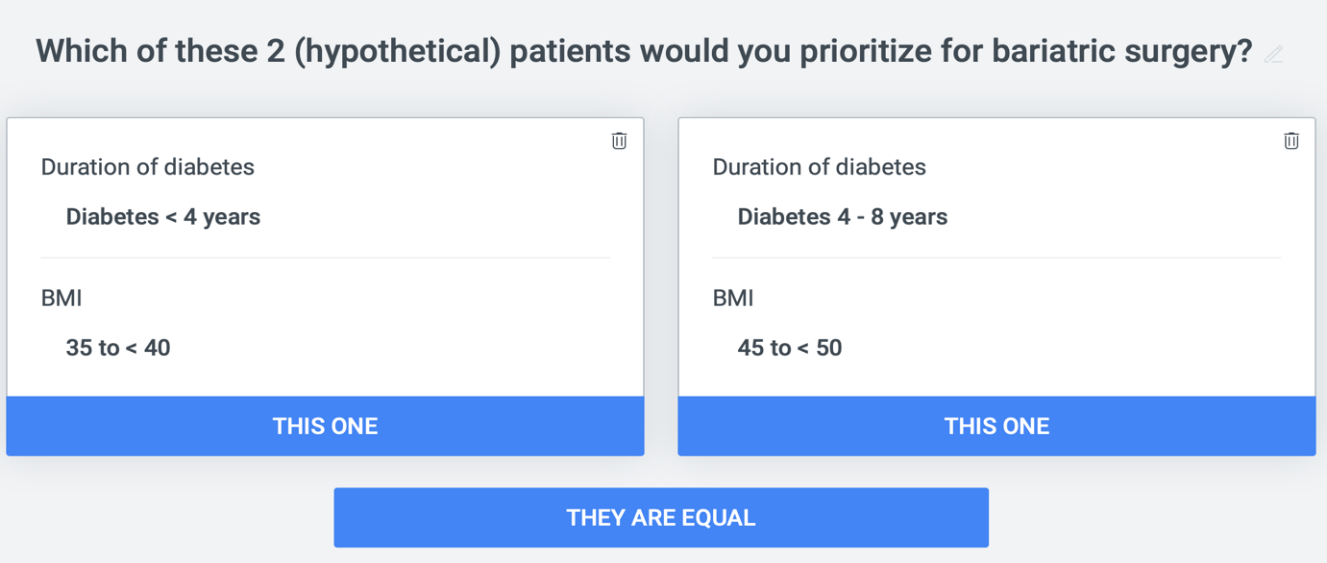


Figure S1. Examples of participant choices during development of the BAPT scoring framework

Scoring was developed using the web-based 1000Minds software (www.1000minds.com).


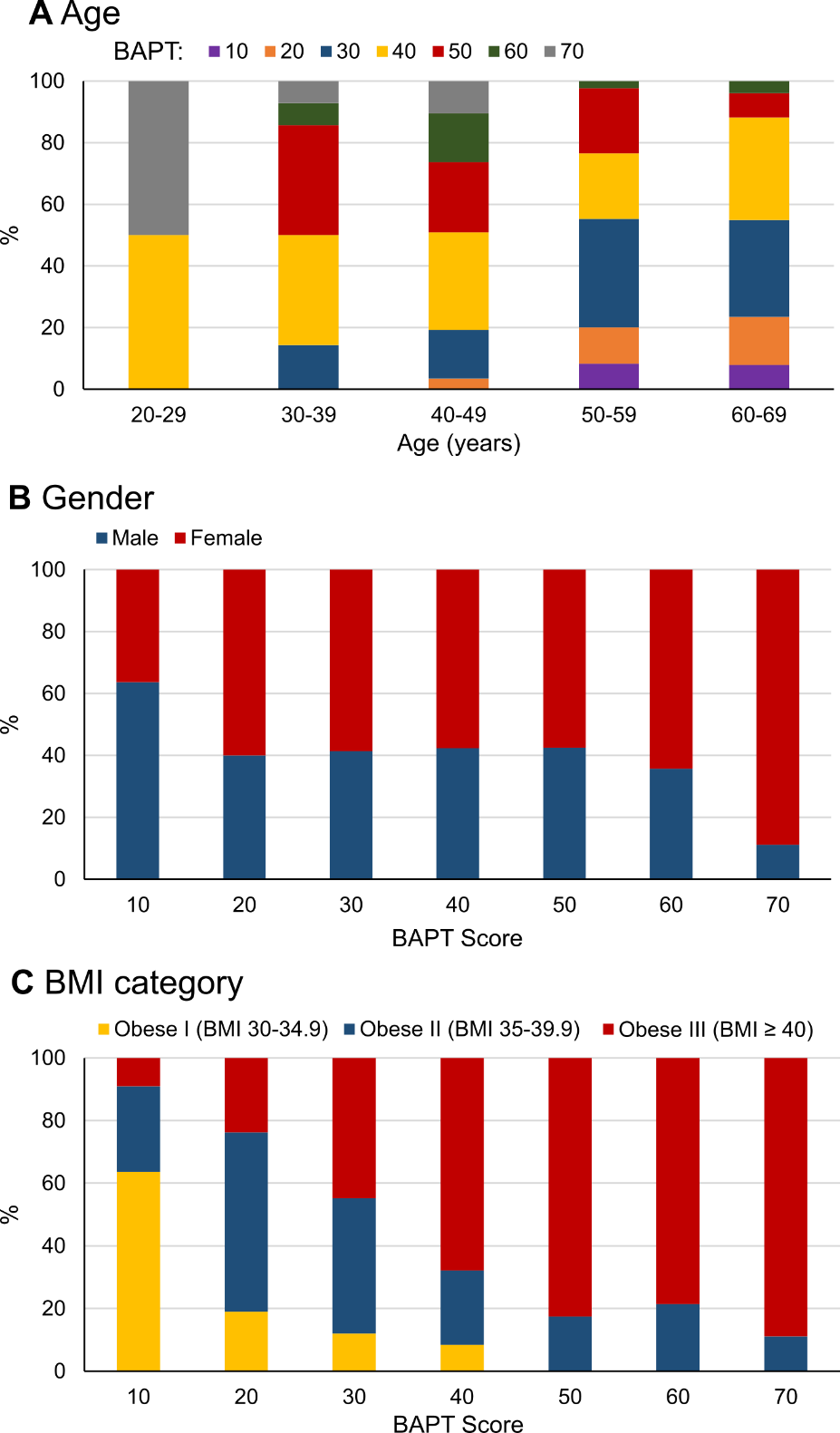


Figure S2. Patient age, BMI and sex distribution across BAPT scores

Patients (N = 292) were grouped according to their BAPT scores. Those with scores of 10–19 are in BAPT Score 10; those with 20–29 are in BAPT Score 20, and so on. The number of patients in each BAPT score group from 10 to 70 was 13, 34, 77, 74, 56, 27, and 11, respectively.

Table S1. Adverse events

| **BAPT score** | **Number of persons in BAPT category who had surgery** | **Any adverse event**  **(N, %)** | **Total adverse events**  **(N, %)** |
| --- | --- | --- | --- |
| 10 | 11 | 2 (18.2%) | 2 (18.2%) |
| 20 | 21 | 0 (0.0%) | 0 (0.0% |
| 30 | 59 | 8 (13.6%) | 11 (18.6%) |
| 40 | 59 | 6 (10.2%) | 7 (11.9%) |
| 50 | 39 | 9 (23.1%) | 9 (23.1%) |
| 60 | 14 | 3 (21.4%) | 3 (21.4%) |
| 70 | 9 | 0 (0.0%) | 0 (0.0%) |
|  |  | p=0.962 | p=0.894 |
|  |  |  |  |
| **BAPT surgical risk domain score*** | **Number of persons in BAPT category who had surgery** | **Any Adverse event**  **(N, %)** | **Total Adverse events**  **(N, %)** |
| 0 | 10 | 1 (10.0%) | 1 (10.0%) |
| 10 | 137 | 19 (13.9%) | 22 (16.1%) |
| 20 | 65 | 8 (12.3%) | 9 (13.8% |
|  |  | p=0.584 | p=0.559 |

* BAPT domain scores for surgical risk: 0-1 risk factor = 20 points; 2-3 risk factors = 10 points; 4-5 risk factors = 0 points.

There were 32 adverse events reports in 28 patients; the most common were being readmitted within 28 days (n=15), nausea/vomiting (n=7), returned to theatre (n=3), anastomotic leak (n=3), other surgical complication (n=2), ICU admission (n=1) and infection (n=1).

Table S2. Comparison of quality of life outcomes (AQoL-4D) across BAPT score, pre and post-surgery (mean ± SD)

|  | BAPT score | | | | | | | |
| --- | --- | --- | --- | --- | --- | --- | --- | --- |
| **Quality of Life measures**  (AQoL-4D) | **BAPT score**  10-19 | BAPT score 20-29 | **BAPT score**  30-39 | **BAPT score**  40-49 | **BAPT score**  50-59 | BAPT score 60-69 | BAPT score 70-79 | p-value |
| N | 7 | 7 | 25 | 25 | 21 | 9 | 2 |  |
| Independent living |  |  |  |  |  |  |  |  |
| pre-surgery | 0.971 ± 0.071 | 0.854 ± 0.183 | 0.838 ± 0.246 | 0.806 ± 0.185 | 0.902 ± 0.134 | 0.839 ± 0.217 | 0.847 ± 0.217 | 0.678 |
| 6-months | 1.000 ± 0.000 | 0.866 ± 0.163 | 0.900 ± 0.183 | 0.941 ± 0.105 | 0.920 ± 0.130 | 0.913 ± 0.190 | 0.959 ± 0.072 | 0.956 |
| 12-months | 1.000 ± 0.000 | 0.910 ± 0.143 | 0.900 ± 0.193 | 0.890 ± 0.154 | 0.979 ± 0.067 | 0.938 ± 0.096 | 0.938 ± 0.088 | 0.677 |
| Relationships |  |  |  |  |  |  |  |  |
| pre-surgery | 0.809 ± 0.136 | 0.884 ± 0.131 | 0.836 ± 0.209 | 0.839 ± 0.161 | 0.824 ± 0.207 | 0.714 ± 0.210 | 0.949 ± 0.072 | 0.471 |
| 6-months | 0.949 ± 0.061 | 0.898 ± 0.130 | 0.883 ± 0.171 | 0.869 ± 0.130 | 0.899 ± 0.215 | 0.794 ± 0.280 | 0.966 ± 0.059 | 0.370 |
| 12-months | 0.882 ± 0.121 | 0.851 ± 0.225 | 0.864 ± 0.221 | 0.844 ± 0.210 | 0.923 ± 0.203 | 0.870 ± 0.149 | 1.000 ± 0.000 | 0.431 |
| Senses |  |  |  |  |  |  |  |  |
| pre-surgery | 0.947 ± 0.052 | 0.920 ± 0.097 | 0.908 ± 0.100 | 0.872 ± 0.117 | 0.904 ± 0.088 | 0.906 ± 0.092 | 0.941 ± 0.083 | 0.462 |
| 6-months | 0.980 ± 0.031 | 0.894 ± 0.140 | 0.928 ± 0.083 | 0.914 ± 0.098 | 0.937 ± 0.069 | 0.921 ± 0.109 | 0.980 ± 0.034 | 0.937 |
| 12-months | 0.934 ± 0.090 | 0.920 ± 0.050 | 0.920 ± 0.080 | 0.927 ± 0.077 | 0.945 ± 0.063 | 0.920 ± 0.072 | 1.00 ± 0.00 | 0.395 |
| Mental health |  |  |  |  |  |  |  |  |
| pre-surgery | 0.863 ± 0.108 | 0.819 ± 0.129 | 0.817 ± 0.190 | 0.802 ± 0.135 | 0.794 ± 0.117 | 0.812 ± 0.092 | 0.946 ± 0.076 | 0.730 |
| 6-months | 0.908 ± 0.112 | 0.843 ± 0.128 | 0.847 ± 0.197 | 0.857 ± 0.135 | 0.884 ± 0.117 | 0.895 ± 0.103 | 0.913 ± 0.056 | 0.463 |
| 12-months | 0.876 ± 0.131 | 0.800 ± 0.117 | 0.868 ± 0.137 | 0.855 ± 0.126 | 0.903 ± 0.110 | 0.849 ± 0.112 | 0.923 ± 0.011 | 0.380 |
| Total score |  |  |  |  |  |  |  |  |
| pre-surgery | 0.657 ± 0.149 | 0.582 ± 0.271 | 0.578 ± 0.270 | 0.513 ± 0.227 | 0.561 ± 0.224 | 0.474 ± 0.205 | 0.760 ± 0.339 | 0.414 |
| 6-months | 0.845 ± 0.111 | 0.627 ± 0.231 | 0.657 ± 0.275 | 0.674 ± 0.231 | 0.709 ± 0.240 | 0.669 ± 0.309 | 0.834 ± 0.093 | 0.973 |
| 12-months | 0.738 ± 0.196 | 0.590 ± 0.247 | 0.654 ± 0.274 | 0.633 ± 0.259 | 0.782 ± 0.218 | 0.671 ± 0.251 | 0.870 ± 0.082 | 0.256 |
| AQoL-4D change pre-surgery to 12-months post-surgery *  Mean ± SD (n) | 0.144 ± 0.131 (5) | -0.002 ± 0.476 (4) | 0.045 ± 0.262 (15) | 0.155 ± 0.242 (17) | 0.200 ± 0.246 (15) | 0.137 ± 0.238 (5) | 0.292 ± 0.000 (1) | 0.184 |
| p-value | 0.253 | 0.991 | 0.676 | 0.059 | 0.032 | 0.325 | N/A |  |

* Analysis of complete cases only; Bold numerals indicate the change in AQoL-4D is greater than the minimal important difference of 0.06 [32]
